# Macrophage‐Mimicking Cellular Nanoparticles Scavenge Proinflammatory Cytokines in Specimens of Patients with Inflammatory Disorders

**DOI:** 10.1002/advs.202401423

**Published:** 2024-06-17

**Authors:** Zhidong Zhou, Nilesh Mukundan, Jiayuan Alex Zhang, You‐Ting Wu, Qiangzhe Zhang, Dan Wang, Ronnie H. Fang, Weiwei Gao, Liangfang Zhang

**Affiliations:** ^1^ Department of Nanoengineering and Chemical Engineering Program University of California San Diego La Jolla CA 92093 USA

**Keywords:** cell membrane coating, cellular nanoparticle, cytokine, inflammatory disorder, neutralization

## Abstract

Effectively neutralizing inflammatory cytokines is crucial for managing a variety of inflammatory disorders. Current techniques that target only a subset of cytokines often fall short due to the intricate nature of redundant and compensatory cytokine networks. A promising solution to this challenge is using cell membrane‐coated nanoparticles (CNPs). These nanoparticles replicate the complex interactions between cells and cytokines observed in disease pathology, providing a potential avenue for multiplex cytokine scavenging. While the development of CNPs using experimental animal models has shown great promise, their effectiveness in scavenging multiple cytokines in human diseases has yet to be demonstrated. To bridge this gap, this study selected macrophage membrane‐coated CNPs (MФ‐CNPs) and assessed their ability to scavenge inflammatory cytokines in serum samples from patients with COVID‐19, sepsis, acute pancreatitis, or type‐1 diabetes, along with synovial fluid samples from patients with rheumatoid arthritis. The results show that MФ‐CNPs effectively scavenge critical inflammatory cytokines, including interleukin (IL)‐6, IL‐8, interferon (IFN)‐γ, and tumor necrosis factor (TNF)‐α, in a dose‐dependent manner. Overall, this study demonstrates MФ‐CNPs as a multiplex cytokine scavenging formulation with promising applications in clinical settings to treat a range of inflammatory disorders.

## Graphical abstract

Macrophage‐mimicking cellular nanoparticles (MФ‐CNPs) are fabricated and evaluated for multiplex scavenging of human proinflammatory cytokines ex vivo. It is demonstrated that MФ‐CNPs can effectively capture IL‐6, IL‐8, IFN‐γ, and TNF‐α in serum samples from patients with COVID‐19, sepsis, acute pancreatitis, and type‐1 diabetes, as well as synovial fluid samples from patients with rheumatoid arthritis.

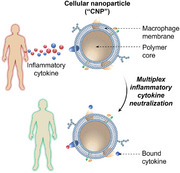

## 1 Introduction

In response to tissue damage, inflammation is crucial in reinstating homeostasis.^[^ ^1^, ^2^ ^]^ Proinflammatory cytokines, secreted by macrophages and dendritic cells (DCs), act as critical mediators, orchestrating innate and subsequent adaptive immune responses to regulate inflammatory reactions.^[^ ^3^ ^]^ The inflammatory process is normally self‐resolving, facilitated by the release of endogenous anti‐inflammatory cytokines and the accumulation of intracellular negative regulatory factors. However, under specific pathophysiological conditions, an uncontrolled release of proinflammatory cytokines occurs, elevating circulating cytokine levels and potentiating immune cell hyperactivation.^[^ ^4^, ^5^ ^]^ This aberrant immune response leads to tissue and organ damage, ultimately underpinning the mortality and morbidity associated with various inflammatory disorders, including sepsis, viral infections, autoimmune diseases, and cancers.^[^ ^6^, ^7^, ^8^, ^9^, ^10^ ^]^

Recognizing the pivotal roles of proinflammatory cytokines in the development and pathogenesis of inflammatory disorders, researchers have dedicated substantial efforts to targeting these cytokines for therapeutic purposes.^[^ ^11^, ^12^ ^]^ In this context, small‐molecule cytokine inhibitors and neutralizing antibodies have demonstrated significant potential, with some achieving notable success.^[^ ^12^, ^13^, ^14^ ^]^ However, suppressing one or a few proinflammatory cytokines does not consistently yield favorable therapeutic outcomes.^[^ ^15^ ^]^ This challenge is primarily attributed to the activation of redundant and compensatory pathways triggered by cytokine signaling, necessitating a comprehensive yet often impractical array of inhibitory agents for effective blockade.^[^ ^16^, ^17^ ^]^ The broad application of free antibodies is also hindered by lack of disease‐specific targeting, leading to dose‐limiting side effects. Complicating matters such as the need for more precise understanding regarding the timing, site, and mechanisms of cytokine involvement in propagating inflammation further add tremendous complexity to effective cytokine inhibition.^[^ ^18^, ^19^ ^]^ Therefore, novel strategies that address these challenges are highly desirable for managing inflammatory disorders.

Cell membrane‐coated nanoparticles (CNPs) have recently garnered significant attention among various emerging cytokine‐neutralization techniques.^[^ ^20^, ^21^ ^]^ These nanoparticles are fabricated by wrapping natural cell membranes onto synthetic nanoparticle substrates. With cell membrane camouflage, they mimic the source cells for biointerfacing, including binding with proinflammatory cytokines for neutralization.^[^ ^22^ ^]^ In this perspective, the CNP platform shows distinct advantages. For example, due to their unique cell mimicry properties, CNPs leverage inherited source receptors to target cytokines without relying on specific cytokine structures for binding and neutralization cues. This function‐driven mechanism allows CNPs to overcome obstacles posed by the vast diversity of targets, a hurdle that has traditionally impeded target structure‐based anti‐cytokine drug development including small‐molecule cytokine inhibitors and neutralizing antibodies. In addition, acting as a decoy of the source cell, CNPs capture cytokines by accurately replicating the complexity and multiplicity of cytokine‐cell receptor binding observed in disease pathology. This capability holds the potential for significantly enhanced efficacy in treatment outcomes.^[^ ^23^ ^]^ Furthermore, CNPs can escape immune clearance, extend systemic circulation time, and target therapeutic sites.^[^ ^20^, ^21^ ^]^ These distinct features make CNPs a unique function‐driven and multiplex cytokine‐neutralizing platform.

Among several CNP formulations, macrophage membrane‐coated CNPs (MФ‐CNPs) stand out for their ability to scavenge and neutralize proinflammatory cytokines, leveraging the prominent roles played by macrophages in cytokine signaling.^[^ ^24^, ^25^, ^26^ ^]^ For instance, in mouse models of sepsis, MФ‐CNPs demonstrated concurrent binding and neutralization of endotoxins and proinflammatory cytokines, thereby inhibiting the cytokine cascade that would otherwise lead to significant mortality.^[^ ^27^ ^]^ MФ‐CNPs specifically inhibited the overactivation of Kupffer cells (KCs), neutralizing proinflammatory cytokines by disrupting the endotoxin‐mediated TLR4/MyD88/IRAK1/NF‐κB signaling pathway.^[^ ^28^ ^]^ This intervention significantly alleviated hepatic ischemia‐reperfusion injury in a rat model of liver transplantation. Moreover, by neutralizing proinflammatory cytokines, MФ‐CNPs were found to suppress inflammation and promote M2 macrophage polarization, facilitating bone tissue repair in a mouse model of femoral defects.^[^ ^29^ ^]^ In combating SARS‐CoV‐2 infection, MФ‐CNPs not only inhibited the entry of the coronavirus into host cells but also neutralized multiple proinflammatory cytokines. As a result, they suppressed macrophage and neutrophil activation, achieving both antiviral and anti‐inflammatory effects.^[^ ^30^, ^31^, ^32^ ^]^ Applying similar principles, MФ‐CNPs have demonstrated efficacy in neutralizing cytokines in animal models of rheumatoid arthritis, atherosclerosis, bone infections, and hyperuricemia, leading to favorable outcomes in treating these diseases.^[^ ^33^, ^34^, ^35^, ^36^, ^37^ ^]^

**Figure 1:**
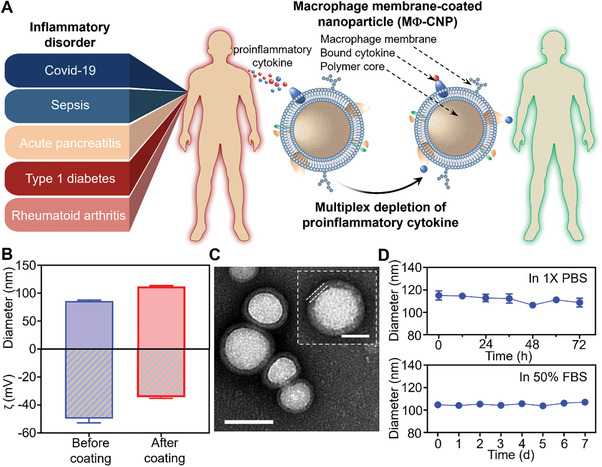
Multiplex cytokine depletion of MΦ‐CNPs in ex vivo specimens of patients with inflammatory disorders including Covid‐19, sepsis, acute pancreatitis, diabetes, and rheumatoid arthritis. A) Schematic illustration showing the use of MΦ‐CNPs to scavenge various proinflammatory cytokines in a wide range of human specimens. B) Hydrodynamic size (diameter, nm) and surface zeta‐potential (ζ, mV) of MΦ‐CNPs measured with dynamic light scattering (*n* = 3; mean + SD). C) Examining MΦ‐CNP morphology with transmission electron microscopy (TEM). Samples were negatively stained with uranyl acetate before imaging. Scale bar = 100 nm. (Insert: a zoomed‐in image of a single MΦ‐CNP; scale bar = 50 nm). D) Testing the stability of MΦ‐CNPs in 1 x phosphate‐buffered saline (PBS) and 50% fetal bovine serum (FBS) by measuring the size change over 72 h and 7 days, respectively (*n* = 3; mean ± SD).

So far, the potential of MФ‐CNPs has only been demonstrated in neutralizing proinflammatory cytokines in animal disease models. This limitation motivates us to explore similar capabilities in a context more pertinent to human diseases. To do so, we obtained sera or synovial fluid specimens from patients with distinct inflammatory disorders, including COVID‐19, sepsis, acute pancreatitis (AP), and type 1 diabetes (T1D), or rheumatoid arthritis (RA) (**Figure** **1**A). Using THP‐1 cells, a human leukemia monocytic cell line, we prepared CNPs accordingly and investigated the ability of these MФ‐CNPs to scavenge proinflammatory cytokines in the above clinical specimens. Our focus centered on four cytokines, including interleukin (IL)−6, IL‐8, interferon‐gamma (IFN‐γ), and tumor necrosis factor‐alpha (TNF‐α), due to their prevalent and prominent roles in these diseases.^[^ ^38^, ^39^, ^40^, ^41^ ^]^ The results demonstrated that MФ‐CNPs effectively scavenged these cytokines in all specimens in a dose‐dependent manner. This study bridges the gap between in vivo animal models and human clinical trials, providing substantial ex vivo pharmacological data to bolster further research and facilitate the translation of CNPs for the treatment of human inflammatory disorders.

## 2 Results and Discussion

### 2.1 Fabrication and Characterization of MФ‐CNPs

We cultured THP‐1 cells and isolated their membranes using a previously established homogenization and centrifugation method.^[^ ^42^ ^]^ Simultaneously, polymeric cores composed of poly(lactic‐co‐glycolic acid) (PLGA) were fabricated using a nanoprecipitation technique.^[^ ^43^ ^]^ Subsequently, we applied a sonication method to coat the THP‐1 membrane onto the PLGA cores. Recent CNP development has led to membrane coating onto nanoparticle cores made of various types of materials.^[^ ^20^, ^21^ ^]^ Among them, PLGA is an attractive material for developing CNP platforms mainly because of its well‐documented biodegradability and biocompatibility that can ease clinical translation.^[^ ^44^, ^45^ ^]^ As depicted in Figure 1B, the initial size of the PLGA core was 86.1 nm ± 1.6 nm. Following the coating process, the size increased to 112.1 nm ± 0.9 nm. Additionally, there was a shift in zeta potential values from −49.5 mV ± 3.0 mV before coating to −34.4 mV ± 0.8 mV after coating. Transmission electron microscopy (TEM) analysis illustrated a core‐shell structure with a uniform size distribution (Figure 1C). Comparing the TEM images of nanoparticle core without membrane coating and MФ‐CNPs clearly revealed the membrane coating (Figure S1, Supporting Information). These findings align with prior CNP formulations and characterizations, confirming the successful MФ‐CNP fabrication.^[^ ^27^ ^]^ Storage experiments in 1X phosphate‐buffered saline (PBS) at 4 °C for 7 days or 50% fetal bovine serum (FBS) at 37 °C for 72 h revealed negligible changes in size for the MФ‐CNP samples, demonstrating robust buffer storage stability and biostability (Figure 1D).

### 2.2 Binding Capacity and Kinetics of MΦ‐CNPs Against Purified Proinflammatory Cytokines

**Figure 2:**
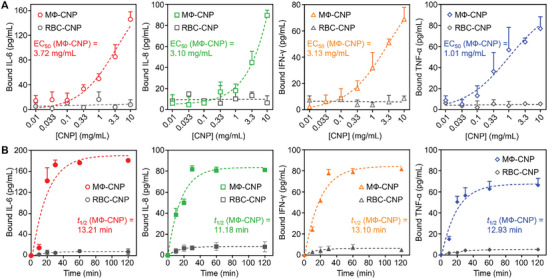
Binding capacity and kinetics of MФ‐CNPs against purified proinflammatory cytokines including IL‐6, IL‐8, IFN‐γ and TNF‐α. A) Determining the binding capacity of MФ‐CNPs with purified cytokines. The cytokine input was held constant while varying the concentration of MФ‐CNPs. B) Investigating the binding kinetics of MФ‐CNPs with purified cytokines. The inputs of cytokines and MФ‐CNPs were fixed, and the levels of bound cytokines were measured at different time points.

We initially investigated cytokine scavenging by assessing the binding efficacy of MΦ‐CNPs with IL‐6, IL‐8, IFN‐γ, and TNF‐α. The binding capacity was assessed by plotting the quantity of bound cytokines against the logarithm of MΦ‐CNP input, revealing a distinctive sigmoid binding profile with evident concentration dependence (**Figure** **2**A). In contrast, the control group using red blood cell membrane‐coated CNPs (RBC‐CNPs, with equivalent membrane loading capacity to MΦ‐CNP, Figure S2, Supporting Information) exhibited minimal cytokine binding. These findings facilitated the determination of MΦ‐CNP concentrations required to scavenge half of the cytokines (EC50), measured at 3.72, 3.10, 3.13, and 1.01 mg mL^−1^ for IL‐6, IL‐8, IFN‐γ, and TNF‐α, respectively. Furthermore, incubating MΦ‐CNPs with antibodies against these cytokines significantly inhibited cytokine binding capacity, demonstrating the binding specificity of MΦ‐CNPs (Figure S3, Supporting Information). The observed cytokine‐scavenging effects of MΦ‐CNPs are attributed to the binding affinities of these cytokines with their corresponding receptors present on the macrophage cell membrane.^[^ ^38^, ^39^, ^40^, ^41^, ^46^ ^]^

In a subsequent experiment investigating the kinetic profiles of CNP‐cytokine binding at specific time points, a time‐dependent binding pattern emerged with MΦ‐CNPs, where the kinetic profiles closely resembled each other (Figure 2B). The levels of bound cytokines increased rapidly before reaching a plateau. Conversely, cytokines exhibited minimal binding with RBC‐CNPs. Applying a pseudo‐first‐order kinetics model to fit the curves, we determined the average rate constants (K) for IL‐6, IL‐8, IFN‐γ, and TNF‐α as 0.05, 0.06, 0.05, and 0.06 min⁻¹, respectively. Based on these data, we calculated that MΦ‐CNPs will achieve 50% binding saturation (*t*_1/2_) with IL‐6, IL‐8, IFN‐γ, and TNF‐α at ≈13.2, 11.2, 13.1, and 11.6 min, respectively.

### 2.3 MΦ‐CNPs Scavenge Proinflammatory Cytokines in COVID‐19 Patient Sera

COVID‐19 is caused by the severe acute respiratory syndrome coronavirus 2 (SARS‐CoV‐2), and its pandemic has led to a global public health crisis.^[^ ^47^ ^]^ This virus predominantly impacts the respiratory tract, often resulting in pneumonia for most patients and acute respiratory distress syndrome (ARDS) in a significant portion of cases. ARDS is the leading cause of mortality in individuals with COVID‐19, primarily triggered by heightened levels of proinflammatory cytokines, a phenomenon known as cytokine storm.^[^ ^48^ ^]^ Key cytokines, including IL‐6 and TNF‐α, play a pivotal role in inflicting substantial lung damage in ARDS patients by impairing the respiratory epithelium, underscoring the potential of multiplex cytokine neutralization in treating COVID‐19.^[^ ^49^ ^]^

**Figure 3:**
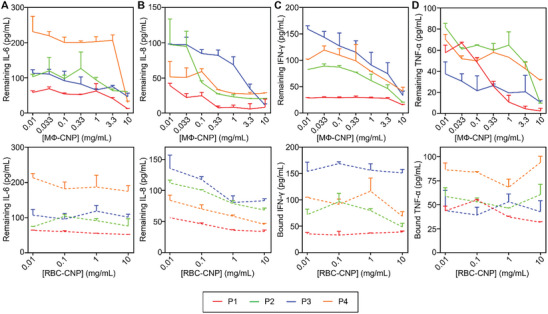
MФ‐CNPs scavenging cytokines in human serum samples from four COVID‐19 patients. The investigated cytokines include A) IL‐6, B) IL‐8, C) IFN‐γ, and D) TNF‐α. RBC‐CNPs were utilized as control groups. Each data point represents the neutralization of serum samples from the same patient with three distinct batches of CNPs.

To assess the effectiveness of MΦ‐CNP in scavenging proinflammatory cytokines present in COVID‐19 patients, we introduced these CNPs into the patients' serum samples. After incubation and removal of the nanoparticles, we quantified the remaining cytokines in the sera. As shown in **Figure** **3**A, the initial levels of IL‐6 varied among different patients. However, as the concentration of MΦ‐CNPs increased, IL‐6 levels decreased proportionally. Despite the differences in IL‐6 levels among the patient samples, MΦ‐CNPs scavenged a significant portion of the cytokine, ranging from 39.2% ± 1.0% to 87.2% ± 1.0% (Table S1, Supporting Information). Conversely, adding RBC‐CNPs to the serum samples had minimal impact on IL‐6 levels, as the cytokine levels showed insignificant changes at various RBN‐CNP concentrations. These findings were consistent for IL‐8, IFN‐γ, and TNF‐α binding (Figure 3B–D). At a 10 mg mL^−1^ concentration, MΦ‐CNPs significantly reduced cytokine levels by varying percentages. In contrast, RBC‐CNPs were ineffective in reducing the serum levels of these cytokines.

### 2.4 MΦ‐CNPs Scavenge Proinflammatory Cytokines in Sepsis Patient Sera

Sepsis is a life‐threatening disease primarily caused by bacterial infections that release the endotoxin lipopolysaccharide (LPS). The interaction of LPS with toll‐like receptor 4 (TLR4) on macrophages activates the nuclear factor‐kappa B (NF‐κB) transcription factor, leading to the release of proinflammatory cytokines such as IL‐6, IFN‐γ, and TNF‐α. The release of these cytokines may induce a cascade effect, triggering an auto‐amplifying cytokine production or cytokine storm, ultimately causing life‐threatening damage to cells and organs.^[^ ^50^ ^]^ MΦ‐CNPs exploit the common functionality of endotoxin or cytokine binding to macrophages, providing a universal neutralization approach across different bacterial genera, species, and strains.^[^ ^27^ ^]^ This nanomedicine design represents a promising biomimetic detoxification strategy that could potentially improve the clinical outcomes of sepsis patients.

**Figure 4:**
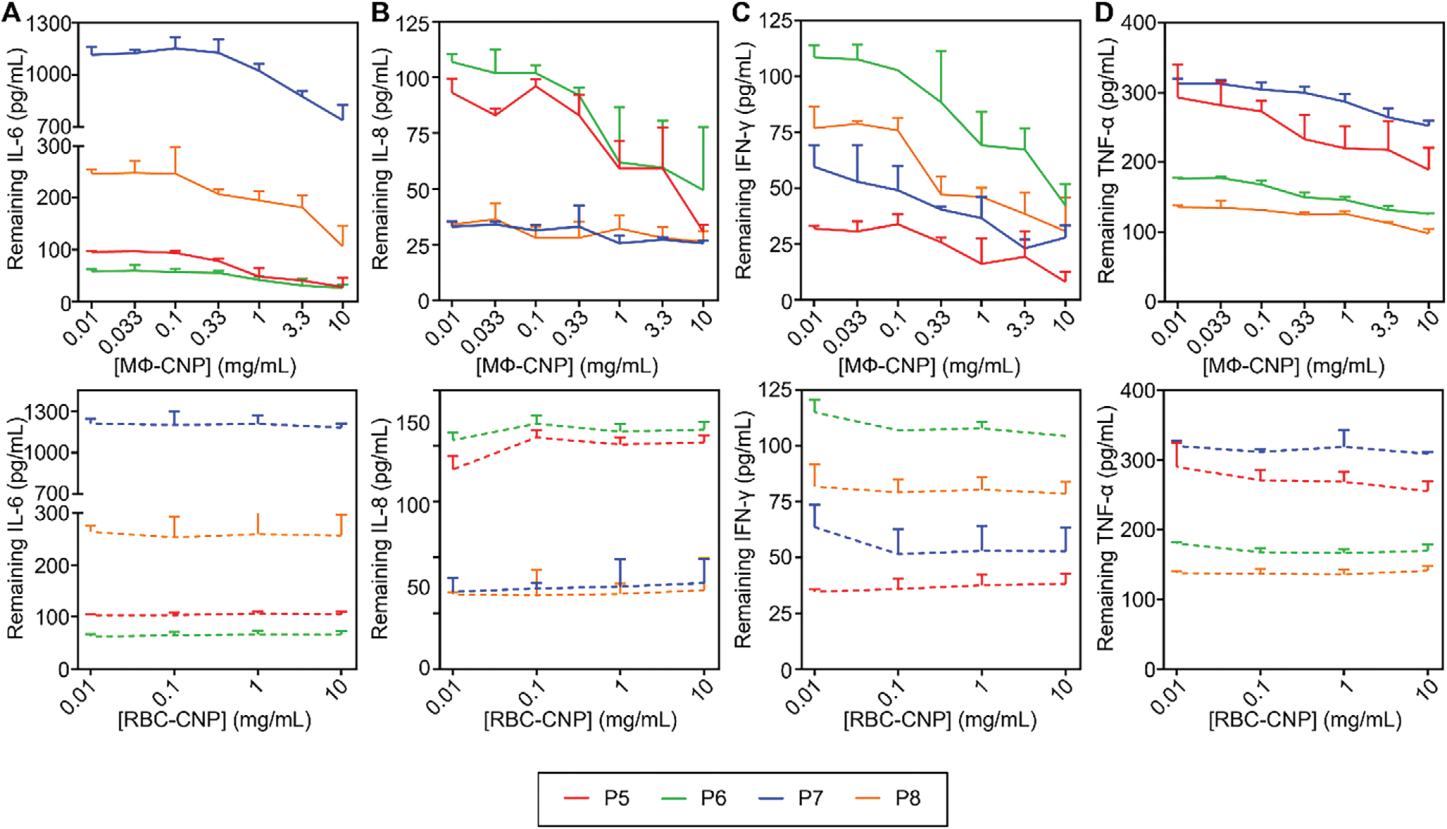
MФ‐CNPs scavenging cytokines in human serum samples from four sepsis patients. The investigated cytokines include A) IL‐6, B) IL‐8, C) IFN‐γ, and D) TNF‐α. RBC‐CNPs were utilized as control groups. Each data point represents the neutralization of serum samples from the same patient with three distinct batches of CNPs.

To evaluate the efficiency of cytokine removal, we obtained the serum samples from sepsis patients and mixed them with MΦ‐CNPs (0.01–10 mg mL^−1^ with half‐log increments) or RBC‐CNPs (0.01–10 mg mL^−1^ with log increments). The mixtures underwent incubation and subsequent centrifugation, as previously described. Enzyme‐linked immunosorbent assay (ELISA) assays were then conducted to quantify the remaining cytokine levels. As illustrated in **Figure** **4**A, an increase in the MΦ‐CNP concentration resulted in a corresponding decrease in IL‐6 levels, indicating a dose‐dependent depletion of IL‐6. At 10 mg mL^−1^, MΦ‐CNPs reduced IL‐6 levels by 70.4% ± 12.4%, 54.4% ± 2.5%, 33.8% ± 2.9%, and 56.6% ± 5.5% for the respective human serum samples (Table S2, Supporting Information). The ability of MΦ‐CNPs to scavenge cytokines was also observed for IL‐8, IFN‐γ, and TNF‐α, despite variations in the initial levels of these cytokines among individual patients (Figure 4B–D). In contrast, RBC‐CNP controls exhibited insignificant cytokine‐scavenging effects.

### 2.5 MΦ‐CNPs Scavenge Inflammatory Cytokines in AP Patient Sera

AP is a disease characterized by the premature activation of digestive enzymes in pancreatic acinar cells (PACs), leading to the self‐digestion of the pancreas itself.^[^ ^51^ ^]^ This self‐digestive condition triggers inflammation, edema, hemorrhage, and necrosis of the pancreatic tissue. Injured PACs release various proinflammatory factors, contributing to peripancreatic tissue destruction and damage to distant organs. The primary cause of death is multisystem organ failure resulting from systemic inflammatory response syndrome or sepsis induced by infections in necrotic tissues. Evidence suggests that activated pancreatic macrophages release proinflammatory cytokines in response to tissue damage.^[^ ^52^ ^]^ During the development of pancreatitis, these cytokines are believed to be responsible for the systemic manifestations of AP and associated distant organ failure.^[^ ^53^ ^]^ Importantly, levels of proinflammatory cytokines, including IL‐6, IL‐8, and TNF‐α, correlate with AP severity.^[^ ^54^ ^]^ Therefore, effective depletion of these cytokines has been an attractive treatment option.^[^ ^55^ ^]^

**Figure 5:**
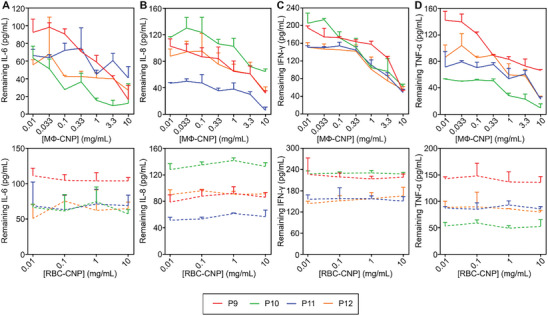
MФ‐CNPs scavenging cytokines in human serum samples from four acute pancreatitis (AP) patients. The examined cytokines include A) IL‐6, B) IL‐8, C) IFN‐γ, and D) TNF‐α. RBC‐CNPs served as control groups. Each data point represents the neutralization of serum samples from the same patient with three distinct CNP batches.

To evaluate the efficiency of proinflammatory cytokine removal, we combined AP patient serum samples with MΦ‐CNPs or RBC‐CNPs at various concentrations, followed by incubation and subsequent nanoparticle removal with centrifugation. The residual cytokines in the supernatant were quantified using ELISA. As shown in **Figure** **5**A, elevating the concentrations of MΦ‐CNPs led to a dose‐dependent reduction in remaining cytokine levels in the sera, signifying a pronounced cytokine scavenging effect. In contrast, RBC‐CNPs exhibited minimal cytokine removal capacity. Treatment of serum samples with 10 mg mL^−1^ of MΦ‐CNPs resulted in substantial reductions, specifically 82.1%, 80.8%, 38.3%, and 51.5% in IL‐6 levels in four AP patient sera, respectively (Table S3, Supporting Information). Furthermore, a similar dose‐dependent reduction in IL‐8, IFN‐γ, and TNF‐α was observed when serum samples were incubated with MΦ‐CNPs but not with RBC‐CNPs, thereby affirming the superior cytokine scavenging capability of MΦ‐CNPs (Figure 5B–D).

### 2.6 MΦ‐CNPs Scavenge Proinflammatory Cytokines in T1D Patient Sera

T1D is a chronic, complex, and multifactorial autoimmune disease in which islet infiltrating immune cells collaborate to destroy insulin‐producing β cells. In this process, proinflammatory cytokines are the significant drivers of inflammation, playing crucial roles in mediating ongoing β‐cell destruction.^[^ ^56^ ^]^ For example, IL‐6 has been shown to sensitize diabetic T cells, potentially promoting autoreactive T cells to traffic to the pancreas for destruction.^[^ ^57^ ^]^ Increased circulating IL‐8 is associated with reduced insulin‐like growth factor 1 (IGF‐1) and poor metabolic control in adolescents with T1D.^[^ ^58^ ^]^ Meanwhile, TNF‐α is directly implicated in the destruction of β‐cells, as observed in in vitro studies on isolated islets.^[^ ^59^ ^]^ In vivo, T1D is associated with elevated serum TNF‐ α levels in both newly diagnosed and poorly controlled patients, indicating a critical role of TNF‐ α in the pathogenesis of the disease.^[^ ^60^ ^]^ Furthermore, IFN‐γ signaling in the islets, including activation of the JAK‐STAT pathway and upregulation of MHC class I, are hallmarks of T1D.^[^ ^61^ ^]^ The complex roles of proinflammatory cytokines in T1D imply that effective cytokine inhibition may restrict effector cell activity, reinforce suppressor cell function, and promote islet recovery from injury.

**Figure 6:**
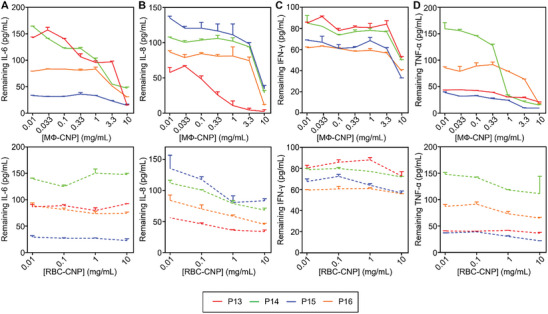
MФ‐CNPs scavenging cytokines in human serum samples from four patients with type 1 diabetes (T1D). The studied cytokines include A) IL‐6, B) IL‐8, C) IFN‐γ, and D) TNF‐α. RBC‐CNPs were employed as control groups. Each data point represents the neutralization of serum samples from identical patients with three separate CNP batches.

**Figure 7:**
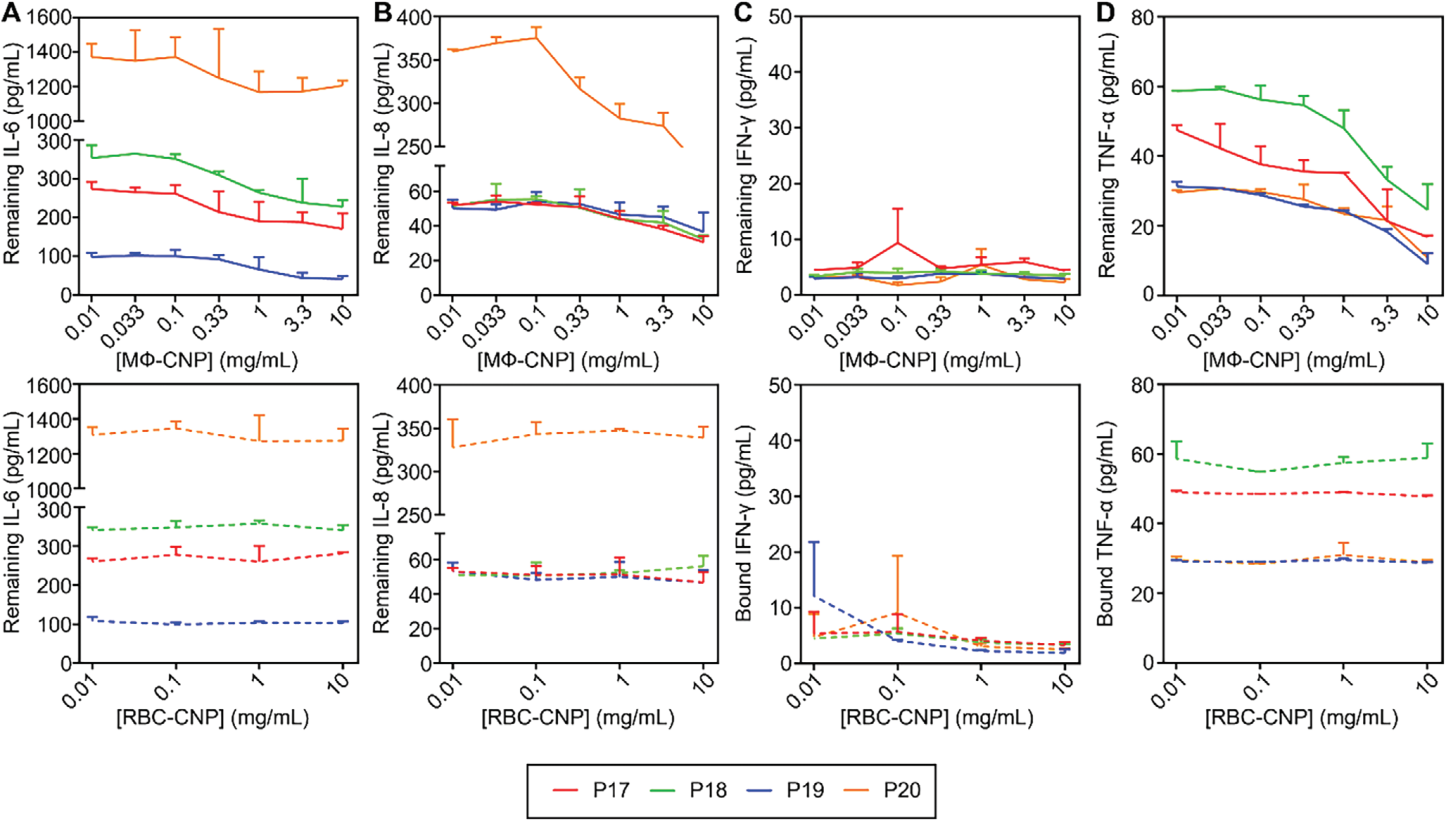
MФ‐CNPs scavenging cytokines in human synovial fluid (SF) samples from four rheumatoid arthritis (RA) patients. The studied cytokines include A) IL‐6, B) IL‐8, C) IFN‐γ, and D) TNF‐α. RBC‐CNPs serve as control groups. Each data point represents the neutralization of serum samples from the same patient with three distinct batches of CNPs.

To test the efficiency of proinflammatory cytokine removal, we mixed serum samples from T1D patients with MΦ‐CNPs (0.01 to 10 mg mL^−1^, half‐log increments). This mixture underwent the identical incubation and nanoparticle removal procedure as mentioned in previous studies, while RBC‐CNPs (0.01–10 mg mL^−1^, log increments) were used as the control group. When the remaining IL‐6 concentration was analyzed, an apparent dose‐dependent depletion profile was shown, whereas RBC‐CNPs displayed insignificant removal of IL‐6 (**Figure** **6**A). At a 10 mg mL^−1^ concentration, MΦ‐CNPs significantly reduced IL‐6 levels for the respective patients, ranging from 56.2% ± 5.0% to 89.9% ± 1.6% (Table S4, Supporting Information). Conversely, the average remaining IL‐6 levels in the serum treated with RBC‐CNPs showed negligible changes. Similar results were indicated for other cytokines, including IL‐8, INF‐γ, and TNF‐α (**Figure** **7**B–D). MΦ‐CNPs significantly reduced the levels of these cytokines. In contrast, the levels of these cytokines remained unchanged when the samples were treated with RBC‐CNPs.

### 2.7 MΦ‐CNPs Scavenge Proinflammatory Cytokines in RA Patient Synovial Fluid Samples

RA is a widespread and debilitating autoimmune disorder characterized by systemic inflammation, resulting in progressive joint deterioration and disability.^[^ ^62^ ^]^ The precise cause of RA remains elusive, prompting current treatments to target the proinflammatory cytokines. While some success has been observed, especially with biologics inhibiting TNF‐α and IL‐1, existing approaches are hindered by significant limitations.^[^ ^63^ ^]^ The orchestration of pathological inflammation in RA involves numerous cytokines and other signaling molecules. Inhibiting one or a few may prove inadequate to impede or reverse disease progression.^[^ ^64^ ^]^ Present cytokine inhibition regimens inadequately control the condition in a significant portion of the patients, with only a minor fraction achieving sustained clinical remission.^[^ ^65^ ^]^ Additionally, the multitude of cytokine targets contributes to the unpredictable toxicity of cytokine inhibition, resulting in notable adverse effects and safety concerns.^[^ ^66^ ^]^ Therefore, it is imperative to develop alternative cytokine inhibition strategies capable of navigating the complexities and variations in the inflammatory network to enhance the effectiveness of RA treatment.

To evaluate the effectiveness of MΦ‐CNPs in depleting proinflammatory cytokines in RA, we mixed the nanoparticles with synovial fluid (SF) samples obtained from RA patients. After incubation and removal of the nanoparticles, we quantified cytokine levels using ELISA. As illustrated in Figure 7A, the cytokine levels in SF samples decreased with increasing concentrations of nanoparticles. Specifically, when the MΦ‐CNP concentration reached 10 mg mL^−1^, the IL‐6 concentration exhibited reductions of 37.7% ± 3.3%, 35.5% ± 1.5%, 57.5% ± 1.8%, and 12.2% ± 1.4% in four RA patient samples, respectively (Table S5, Supporting Information). In contrast, RBC‐CNPs were ineffective in removing the cytokine, confirming the specificity of depletion by MΦ‐CNPs. A similar dose‐dependent cytokine reduction pattern was observed against IL‐8 (Figure 7B). Notably, all patient SF samples exhibited low levels of IFN‐γ, resulting in the absence of reduction by MΦ‐CNPs (Figure 7C). Consistent with the depletion of IL‐6 and IL‐8, MΦ‐CNPs demonstrated dose‐dependent removal of TNF‐α (Figure 7D).

## 3 Conclusion and Outlook

In summary, this study investigates the clinical potential of MΦ‐CNPs to scavenge proinflammatory cytokines in patients with various inflammatory disorders. To achieve this objective, we engineered MΦ‐CNPs by incorporating membranes from human monocytic cells onto PLGA polymeric cores. To assess the cytokine scavenging capabilities of MΦ‐CNPs, we collected serum samples from patients affected by inflammatory conditions such as COVID‐19, sepsis, AP, or T1D. Additionally, SF samples were obtained from RA patients. Utilizing these clinically relevant samples, our investigations revealed that MΦ‐CNPs exerted a significant dose‐dependent reduction in key cytokines, including IL‐6, IL‐8, IFN‐γ, and TNF‐α, compared to the control group treated with RBC‐CNPs. The remarkable ability of MΦ‐CNPs to effectively scavenge proinflammatory cytokines across a wide spectrum of diverse diseases highlights their potential as a universal platform for multiplex cytokine depletion. Utilizing only patient sera in this study to assess MΦ‐CNPs' capacity for neutralizing disease‐associated human cytokines, these preclinical findings illustrate the potential of this nanomedicine formulation for clinical translation.

The application potential of MΦ‐CNPs for cytokine neutralization is increasingly evident, especially with recent advancements in integrating these nanoparticles with existing technology toward enhancing their biointerfacial properties. One notable study demonstrated the enhancement of surface heparin density through a combination of metabolic engineering and surface conjugation techniques on MΦ‐CNPs, resulting in enhanced efficacy against SARS‐CoV‐2.^[^ ^67^ ^]^ This breakthrough not only holds promise for combatting other glycan‐dependent viruses but also underscores the versatility of MΦ‐CNPs in targeted viral inhibition in general. Moreover, leveraging metabolic engineering techniques, researchers successfully enhanced the expression levels of gangliosides on THP‐1 cells, leading to the creation of MΦ‐CNPs with significantly improved efficacy in neutralizing botulinum toxin compared to their unmodified counterparts.^[^ ^68^ ^]^ This study highlights the potential of MΦ‐CNPs, particularly when engineered with glycans, as a versatile platform for counteracting neurotoxins. Further advancements through genetic engineering resulted in THP‐1 cells expressing proline‐alanine‐serine (PAS) peptide chains.^[^ ^69^ ^]^ Incorporating these PAS chains into MΦ‐CNPs conferred protection against opsonization and phagocytosis, prolonging their residence times following intravenous or intratracheal administration beyond those coated with wild‐type membranes. Consequently, these modified MΦ‐CNPs exhibited enhanced efficacy in inhibiting inflammatory cytokines in murine models of lipopolysaccharide‐induced lung injury and sublethal endotoxemia. These are exciting findings and progress in developing MΦ‐CNPs, not only contributing to our understanding of MΦ‐CNPs but also paving the way for their further development and eventual clinical translation as a promising biomimetic strategy for treating inflammatory conditions characterized by cytokine dysregulation.

## 4 Experimental Section

### Macrophage Culture and Membrane Derivation

The human THP‐1 cell line (TIB‐202, ATCC) was cultured in RPMI 1640 (Gibco) supplemented with 10% fetal bovine serum (FBS, Hyclone) and 1% penicillin‐streptomycin (Gibco). Cells were maintained at 37 °C in a humidified incubator with 5% CO2 and were regularly tested for mycoplasma contamination every 2 weeks. The plasma membrane of THP‐1 cells was derived following a previously published protocol.^[^ ^42^ ^]^ Briefly, THP‐1 cells were cultured in suspension flasks (CELLTRAT 850 mL suspension flask) or spinner flasks (Wilmad Labglass) to a density of 2 × 10^6^ cells mL^−1^ and washed three times in 1X PBS with centrifugation (800 ×g, 10 min). After washing, cells were suspended in a hypotonic lysing buffer containing 30 mm Tris‐HCl (pH 7.5), 225 mm D‐mannitol, 75 mm sucrose, 0.2 mm ethylene glycol‐bis(b‐aminoethyl ether)‐N, N, N’, N’‐tetraacetic acid (EGTA), and protease and phosphatase inhibitor cocktails (all from Millipore‐Sigma). Cell homogenization was achieved using a Kinematica Polytron PT‐2000 probe homogenizer. The homogenized solution was then centrifuged at 7600 ×g for 25 min at 4 °C using an ultracentrifuge. After removing the pellet, the supernatant was subjected to a second centrifugation at 29 600 ×g for 35 min at 4 °C. The resulting pellet, containing the membrane, was suspended in 0.2 mm ethylenediaminetetraacetic acid (EDTA) solution and centrifuged again at 29 600 ×g for 35 min at 4 °C. EDTA was used for chelating ions and thus minimizing membrane aggregation. The membrane was collected as the pellet and then resuspended in DI water. The protein content was quantified using a BCA protein assay kit (Thermal Fisher Scientific). The membrane suspension was stored in 0.2 mm EDTA solution at −80 °C for subsequent studies.

### RBC Membrane Derivation

The derivation of RBC membrane followed a previously established protocol.^[^ ^43^ ^]^ Briefly, washed human RBCs (BioIVT) were resuspended in 0.25X PBS in an ice bath for 20 min for hypotonic lysis. Lysed cells were then centrifuged at 20 000 ×g for 10 min. The supernatant containing released hemoglobin was carefully removed, and the pellet was resuspended in 0.25X PBS in an ice bath for 20 min, followed by centrifugation and resuspension using the same process. This cycle was repeated five times to ensure the removal of hemoglobin. The RBC membrane, collected as a pellet with a light pink color, was resuspended in DI water. The protein content was quantified using a BCA protein assay kit (Thermal Fisher Scientific). The suspension was stored at −80 °C for subsequent studies.

### CNP Fabrication and Characterization

To synthesize polymeric nanoparticle cores, 0.4 mL of poly(DL‐lactic‐co‐glycolic acid) (50:50 PLGA, 0.67 dl g^−1^; Lactel Absorbable Polymers) in acetone (20 mg mL^−1^) was rapidly added to 1 mL of water. The solution was subjected to a vacuum aspirator until complete acetone evaporation. For coating, the macrophage or RBC membrane was combined with the PLGA core at a polymer‐to‐membrane protein weight ratio of 1:1. Sonication in a water bath for 2 min facilitated membrane coating onto the core. Subsequently, CNPs were concentrated to a protein concentration of 10 mg mL^−1^ through centrifugation at 4500 ×g for 15 min using an Amicon ultracentrifugal filter (30 kDa MWCO, Millipore‐Sigma). MФ‐CNPs were characterized for hydrodynamic size and surface zeta potential using dynamic light scattering (DLS) with a Malvern ZEN 3600 Zetasizer. To examine the morphology, MФ‐CNPs were stained with uranyl acetate (1 wt%) and visualized using a JEOL JEM‐1400Plus transmission electron microscope at the Cellular and Molecular Medicine Electron Microscopy Core (UCSD‐CMM‐EM Core, RRID: SCR_02 2039). A long‐term colloidal stability test was performed in 1X PBS or 0.5X FBS, maintaining a nanoparticle suspension concentration at a final protein concentration of 0.5 mg mL^−1^. Samples were stored at 37 °C, and hydrodynamic size was measured daily for 7 days.

### CNP Binding with Purified Inflammatory Cytokines

Freshly made MФ‐CNPs were blocked by 1 w/v% bovine serum albumin (BSA) in PBS for 30 min at room temperature. The CNPs with concentrations ranging from 0.01–10 mg mL^−1^ were then mixed with recombinant human IL‐6 (200 pg mL^−1^), IL‐8 (100 pg mL^−1^), IFN‐γ (100 pg mL^−1^), or TNF‐α (100 pg mL^−1^). The mixtures were incubated at 37 °C in a water bath for 2 h. After the incubation, CNPs were removed by centrifugation at 25 000 ×*g* for 15 min, and the supernatant was collected and tested for the concentration of unbound cytokines with ELISA (Biolegend) following manufacturer's instruction. The levels of bound cytokines were calculated by subtracting the concentration of unbound cytokines from initial cytokine levels. As for cytokine binding kinetic study, human IL‐6 (200 pg mL^−1^), IL‐8 (100 pg mL^−1^), IFN‐γ (100 pg mL^−1^), or TNF‐α (100 pg mL^−1^) were mixed with MФ‐CNPs (10 mg mL^−1^). The mixtures were incubated for 10, 20, 30, 60, and 120 min in a 37 °C water bath. At each time point, samples were centrifuged at 25 000 ×g for 15 min, and the supernatant was collected. Concentrations of unbound cytokines were quantified with ELISA assay (Biolegend) following the manufacturer's instruction. The concentration of bound cytokines was calculated by subtracting the concentration of unbound cytokines from the initial cytokine level. The neutralization and binding kinetics were analyzed with GraphPad Prism. Neutralization kinetics were fitted using a variable slope model, while a one‐phase association model fitted binding kinetics.

### Ex Vivo Scavenging of Cytokine in Patient Sera or Synovial Fluid Samples

Serum samples from patients with COVID‐19 were purchased from RayBiotech after deactivation of residual viruses. Serum samples from patients with sepsis, acute pancreatitis, and diabetes were purchased from BioIVT. Synovial fluid samples from patients with osteoarthritis were purchased from BioIVT. Note that all human specimens were purchased from vendors and no patient identity was involved. In the study, all serum samples were diluted 10X before the experimental tests. MФ‐CNPs ranging from 0.01 to 10 mg mL^−1^ were blocked with 1 w/v% BSA in PBS for 30 min and then mixed with serum samples. The mixtures were incubated at 37 °C for 2 h. After the incubation, samples were centrifuged at 25 000 ×*g* for 15 min. The supernatants were taken and cytokine concentrations in the supernatants were quantified with ELISA assay following the manufacturer's instruction. RBC‐CNPs were served as a control group. The percentage of cytokine reduction is defined as the difference between the initial cytokine level in the specimens and the cytokine level after removal by 10 mg mL^−1^ CNPs divided by the initial cytokine level.

## Conflict of Interest

L.Z. is the founder of Cellics Therapeutics and holds equity interest in the company. The other authors declare no conflict of interest.

## Supporting information

Supporting Information

## Data Availability

The data that support the findings of this study are available from the corresponding author upon reasonable request.
