# Supplementary material for: Macrophage‐Mimicking Cellular Nanoparticles Scavenge Proinflammatory Cytokines in Specimens of Patients with Inflammatory Disorders
Source: Adv Sci (Weinh). 2024 Jun 17;11(31):2401423. doi: 10.1002/advs.202401423 (PMC11336921; doi:10.1002/advs.202401423)
Supplement: Supplementary file 1 — Supporting Information [file ADVS-11-2401423-s001.docx]

Supporting Information:

**Macrophage-Mimicking Cellular Nanoparticles Scavenge Proinflammatory Cytokines in Specimens of Patients with Inflammatory Disorders**

Zhidong Zhou, Nilesh Mukundan, Jiayuan Alex Zhang, You-ting Wu, Qiangzhe Zhang, Dan Wang, Ronnie H. Fang, Weiwei Gao*, and Liangfang Zhang*

Department of Nanoengineering and Chemical Engineering Program, University of California San Diego, La Jolla, CA 92093, USA

* Correspondence authors:

Weiwei Gao ([w5gao@ucsd.edu](mailto:w5gao@ucsd.edu))

Liangfang Zhang ([zhang@ucsd.edu](mailto:zhang@ucsd.edu))

**Supporting Figures**

**Supporting Figure S1**


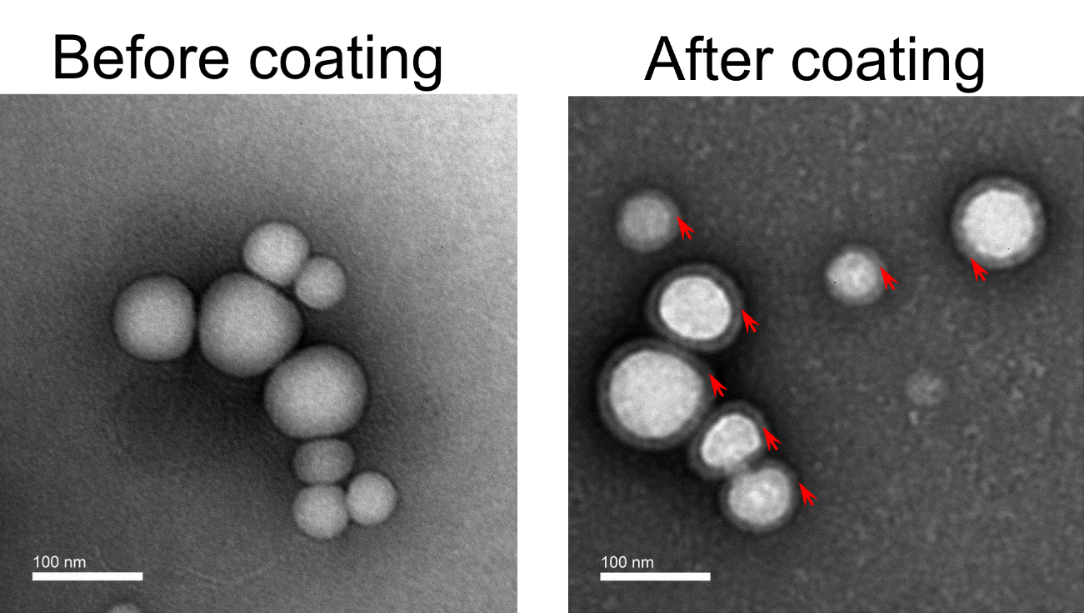


**Figure S1.** Direct visualization of membrane coating. In the study, we compared nanoparticle morphology with transmission electron microscopy (TEM) before and after the membrane coating. Before coating (left), nanoparticles appear as solid spheres. After coating (right), a clear membrane shell appeared around the solid core, indicating the coated membrane. Arrows (red) in the image indicate the membrane shell.

**Supporting Figure S2.**

**
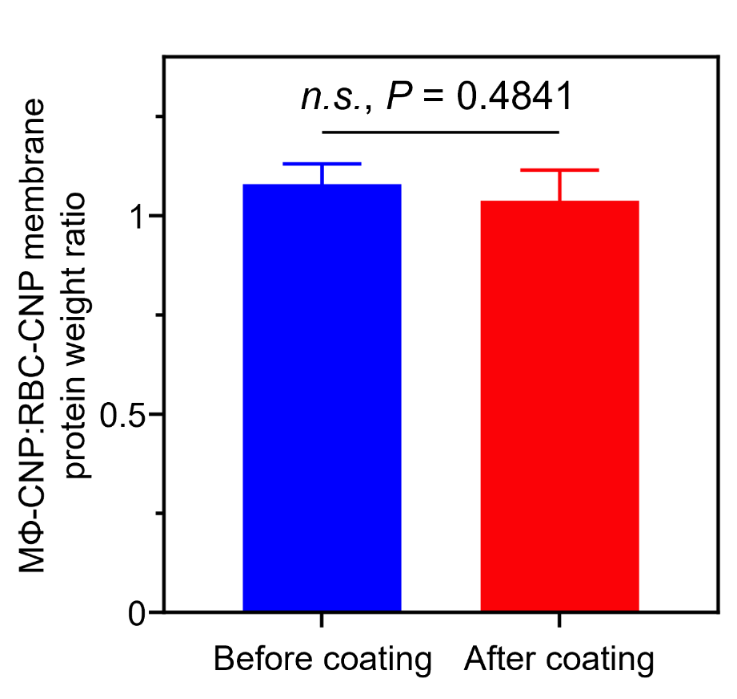
**

**Figure S2.** Comparison of the MΦ and RBC membrane coating efficiency onto the PLGA cores. In this study, MΦ and RBC membranes were coated onto PLGA core, respectively. The membranes inputs were the same before coating, represented by a ratio of approximately 1. After the coating, the nanoparticles were isolated with centrifugation (25 k ×g, 10 min). The protein content measured from both nanoparticles remained the same, indicating comparable coating efficiency. In the study, the membrane content was quantified with a BCA assay. Data presented as mean + s.d. The statistical analysis was performed using the student’s t-test, *n.s*.: not significant.

**Supporting Figure S3**

**
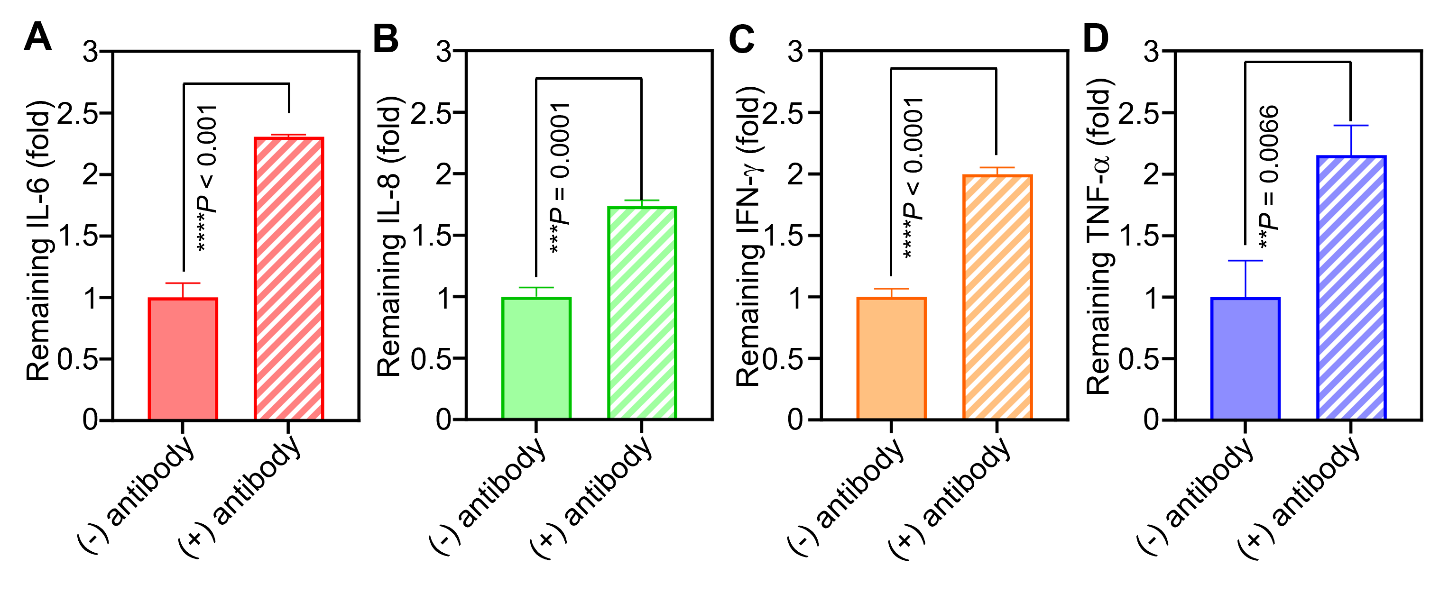
**

**Figure S3.** Measuring remaining cytokine levels in the supernatant after neutralization by using MФ-CNPs. (A) IL-6, (B) IL-8, (C) IFN-γ, and (D) TNF-α were incubated with MФ-CNPs with or without adding cytokine-specific antibodies, including anti-IL-6 (Biolegend, 352801), anti-IL-8 (Biolegend, 320605), anti-IFN-γ (Biolegend, 308606), and anti-TNF-α (Biolegend, 358402), respectively, for 2 h. After the incubation, the MФ-CNPs were spung down. The cytokine level in the remaining supernatant was measured with ELISA. The remaining cytokine levels in the group without blocking antibodies were normalized to 1 and compared with the group with blocking antibodies. In this study, the initial concentrations of IL-6, IL-8, IFN-γ, and TNF-α were 200, 100, 100 and 100 pg/mL, respectively. MФ-CNPs concentration in all groups was 3.33 mg/mL. Statistical analysis was performed using an unpaired student t-test. In all datasets, data are presented as mean + s.d., n = 3 per group.

| **Supporting Tables**  **Table S1.** Cytokine reduction by MΦ-CNPs in COVID-19 patient serum samples | | | | |
| --- | --- | --- | --- | --- |
| Patient | Cytokine | Cytokine level  (pg/mL, [MΦ-CNP] = 0.01 mg/mL) | Cytokine level  (pg/mL, [MΦ-CNP] = 10 mg/mL) | Percent of reduction (%) |
| 1 | IL-6 | 58.8 ± 3.4 | 12.1 ± 0.6 | 79.4 ± 1.2 |
|  | IL-8 | 38.9 ± 2.4 | 8.4 ± 8.1 | 78.5 ± 4.4 |
|  | IFN-γ | 28.6 ± 0.7 | 15.4 ± 0.9 | 46.1 ± 2.3 |
|  | TNF-α | 57.7 ± 5.0 | 2.4 ± 1.8 | 95.9 ± 1.4 |
| 2 | IL-6 | 100.1 ± 7.9 | 60.9 ± 0.0 | 39.2 ± 1.0 |
|  | IL-8 | 97.3 ± 25.7 | 20.6 ± 0.3 | 78.8 ± 1.0 |
|  | IFN-γ | 82.5 ± 0.8 | 20.3 ± 0.4 | 75.5 ± 1.3 |
|  | TNF-α | 81.8 ± 2.5 | 9.4 ± 0.3 | 88.5 ± 1.1 |
| 3 | IL-6 | 113.3 ± 7.2 | 46.7 ± 7.2 | 58.8 ± 2.0 |
|  | IL-8 | 97.3 ± 0.7 | 11.3 ± 1.3 | 88.4 ± 3.0 |
|  | IFN-γ | 159.2 ± 4.3 | 33.3 ± 6.0 | 79.1 ± 2.4 |
|  | TNF-α | 37.6 ± 8.0 | 10.6 ± 1.0 | 71.8 ± 1.1 |
| 4 | IL-6 | 231.3 ± 30.8 | 29.7 ± 3.1 | 87.2 ± 1.0 |
|  | IL-8 | 51.3 ± 15.5 | 28.4 ± 0.4 | 44.7 ± 1.0 |
|  | IFN-γ | 100.9 ± 1.0 | 41.3 ± 5.4 | 59.1 ± 6.2 |
|  | TNF-α | 70.4 ± 3.3 | 31.2 ± 0.8 | 55.6 ± 1.2 |

| **Table S2.** Cytokine reduction by MΦ-CNPs in sepsis patient serum samples | | | | |
| --- | --- | --- | --- | --- |
| Patient | Cytokine | Cytokine level  (pg/mL, [MΦ-CNP] = 0.01 mg/mL) | Cytokine level  (pg/mL, [MΦ-CNP] = 10 mg/mL) | Percent of reduction (%) |
| 5 | IL-6 | 96.1 ± 1.1 | 28.4 ± 12.5 | 70.4 ± 12.4 |
|  | IL-8 | 93.2 ± 4.5 | 30.9 ± 2.2 | 66.9 ± 1.5 |
|  | IFN-γ | 32.0 ± 0.7 | 8.1 ± 3.2 | 74.8 ± 5.5 |
|  | TNF-α | 293.3 ± 33.3 | 189.5 ± 22.3 | 35.4 ± 1.7 |
| 6 | IL-6 | 58.5 ± 3.0 | 26.7 ± 4.6 | 54.4 ± 2.5 |
|  | IL-8 | 107.0 ± 2.5 | 49.5 ± 20.0 | 53.7 ± 9.0 |
|  | IFN-γ | 108.7 ± 3.9 | 42.3 ± 6.7 | 61.1 ± 2.8 |
|  | TNF-α | 176.8 ± 1.2 | 126.1 ± 0.6 | 28.7 ± 1.5 |
| 7 | IL-6 | 1116.1 ± 33.3 | 738.1 ± 62.1 | 33.8 ± 2.9 |
|  | IL-8 | 33.2 ± 1.7 | 25.7 ± 0.8 | 22.6 ± 1.5 |
|  | IFN-γ | 59.6 ± 6.7 | 27.9 ± 3.9 | 53.2 ± 1.6 |
|  | TNF-α | 312.7 ± 5.2 | 252.7 ± 5.2 | 19.2 ± 2.0 |
| 8 | IL-6 | 246.6 ± 6.0 | 107.0 ± 27.3 | 56.6 ± 5.5 |
|  | IL-8 | 34.0 ± 0.8 | 26.5 ± 3.33 | 22.1 ± 5.0 |
|  | IFN-γ | 76.9 ± 6.7 | 30.8 ± 10.9 | 60.0 ± 2.6 |
|  | TNF-α | 135.6 ± 2.0 | 97.1 ± 5.9 | 28.4 ± 3.9 |

| **Table S3.** Cytokine reduction by MΦ-CNPs in acute pancreatitis (AP) patient serum samples | | | | |
| --- | --- | --- | --- | --- |
| Patient | Cytokine | Cytokine level  (pg/mL, [MΦ-CNP] = 0.01 mg/mL) | Cytokine level  (pg/mL, [MΦ-CNP] = 10 mg/mL) | Percent of reduction (%) |
| 9 | IL-6 | 92.5 ± 10.6 | 16.6 ± 11.2 | 82.1 ± 2.1 |
|  | IL-8 | 102.8 ± 7.7 | 31.9 ± 2.6 | 69.0 ± 1.3 |
|  | IFN-γ | 194.9 ± 2.9 | 47.8 ± 5.0 | 75.5 ± 2.7 |
|  | TNF-α | 142.3 ± 9.4 | 66.4 ± 0.9 | 53.4 ± 1.1 |
| 10 | IL-6 | 63.5 ± 9.6 | 12.2 ± 15.7 | 80.8 ± 2.6 |
|  | IL-8 | 115.8 ± 1.0 | 64.4 ± 2.9 | 44.4 ± 4.0 |
|  | IFN-γ | 205.4 ± 16.3 | 61.2 ± 4.5 | 70.2 ± 1.3 |
|  | TNF-α | 53.2 ± 0.5 | 9.8 ± 4.3 | 81.5 ± 10.0 |
| 11 | IL-6 | 66.4 ± 4.9 | 41.0 ± 9.0 | 38.3 ± 2.8 |
|  | IL-8 | 45.9 ± 1.8 | 5.9 ± 3.5 | 87.2 ± 3.0 |
|  | IFN-γ | 150.8 ± 2.5 | 48.4 ± 4.9 | 67.9 ± 3.0 |
|  | TNF-α | 71.6 ± 16.4 | 23.2 ± 2.5 | 67.6 ± 1.2 |
| 12 | IL-6 | 55.7 ± 4.1 | 27.1 ± 4.9 | 51.5 ± 2.2 |
|  | IL-8 | 87.5 ± 7.8 | 33.6 ± 5.5 | 61.6 ± 1.7 |
|  | IFN-γ | 150.8 ± 7.4 | 54.9 ± 4.9 | 63.6 ± 1.7 |
|  | TNF-α | 86.3 ± 13.1 | 23.2 ± 3.3 | 73.1 ± 1.3 |

| **Table S4.** Cytokine reduction by MΦ-CNPs in type 1 diabetes (T1D) patient serum samples | | | | |
| --- | --- | --- | --- | --- |
| Patient | Cytokine | Cytokine level  (pg/mL, [MΦ-CNP] = 0.01 mg/mL) | Cytokine level  (pg/mL, [MΦ-CNP] = 10 mg/mL) | Percent of reduction (%) |
| 13 | IL-6 | 142.5 ± 1.2 | 14.4 ± 0.8 | 89.9 ± 1.6 |
|  | IL-8 | 57.7 ± 5.0 | 2.4 ± 1.8 | 95.9 ± 1.4 |
|  | IFN-γ | 85.1 ± 0.6 | 52.0 ± 1.1 | 38.9 ± 2.8 |
|  | TNF-α | 43.7 ± 0.1 | 21.0 ± 0.1 | 51.9 ± 3.0 |
| 14 | IL-6 | 164.6 ± 0.2 | 47.2 ± 1.7 | 71.3 ± 12.2 |
|  | IL-8 | 107.3 ± 0.6 | 28.8 ± 2.4 | 73.2 ± 5.1 |
|  | IFN-γ | 85.1 ± 5.0 | 49.7 ± 0.5 | 41.6 ± 1.1 |
|  | TNF-α | 159.7 ± 8.2 | 15.2 ± 0.7 | 90.5 ± 1.1 |
| 15 | IL-6 | 33.5 ± 0.5 | 14.7 ± 1.8 | 56.2 ± 5.0 |
|  | IL-8 | 135.0 ± 1.9 | 34.4 ± 3.5 | 74.5 ± 2.8 |
|  | IFN-γ | 68.9 ± 0.1 | 32.8 ± 0.2 | 52.4 ± 3.0 |
|  | TNF-α | 38.8 ± 0.9 | 9.7 ± 0 | 75.1 ± 1.0 |
| 16 | IL-6 | 79.3 ± 0.2 | 30.4 ± 0.5 | 61.7 ± 4.0 |
|  | IL-8 | 86.8 ± 1.4 | 11.0 ± 0.8 | 87.3 ± 1.6 |
|  | IFN-γ | 61.6 ± 0.6 | 39.8 ± 0.9 | 35.4 ± 2.4 |
|  | TNF-α | 83.0 ± 1.8 | 16.9 ± 1.0 | 79.6 ± 1.6 |

| **Table S5.** Cytokine reduction by MΦ-CNPs in rheumatoid arthritis (RA) patient synovial fluid (SF) samples | | | | |
| --- | --- | --- | --- | --- |
| Patient | Cytokine | Cytokine level  (pg/mL, [MΦ-CNP] = 0.01 mg/mL) | Cytokine level  (pg/mL, [MΦ-CNP] = 10 mg/mL) | Percent of reduction (%) |
| 17 | IL-6 | 547.8 ± 25.0 | 341.5 ± 56.3 | 37.7 ± 3.3 |
|  | IL-8 | 52.1 ± 1.1 | 30.8 ± 2.4 | 40.9 ± 3.3 |
|  | IFN-γ | 4.5 ± 0.0 | 4.4 ± 0.1 | 2.9 ± 0.1 |
|  | TNF-α | 47.5 ± 1.1 | 16.8 ± 0.3 | 64.6 ± 1.3 |
| 18 | IL-6 | 707.8 ± 45.0 | 456.5 ± 23.8 | 35.5 ± 1.5 |
|  | IL-8 | 51.8 ± 0.0 | 32.5 ± 1.4 | 37.3 ± 1.4 |
|  | IFN-γ | 3.3 ± 0.1 | 3.5 ± 0.3 | -3.9 ± 3.0 |
|  | TNF-α | 58.7 ± 0.0 | 24.6 ± 5.2 | 58.1 ± 15.0 |
| 19 | IL-6 | 197.8 ± 15.0 | 84.0 ± 11.3 | 57.5 ± 1.8 |
|  | IL-8 | 50.4 ± 3.6 | 36.8 ± 7.9 | 27.0 ± 3.2 |
|  | IFN-γ | 2.9 ± 0.3 | 2.9 ± 0.0 | 0.0 ± 1.0 |
|  | TNF-α | 31.3 ± 0.9 | 9.0 ± 2.2 | 71.2 ± 3.4 |
| 20 | IL-6 | 1372.8 ± 52.5 | 1205.3 ± 20.0 | 12.2 ± 1.4 |
|  | IL-8 | 360.0± 1.8 | 221.4 ± 14.3 | 38.5 ± 9.1 |
|  | IFN-γ | 3.2 ± 0.0 | 2.3 ± 0.4 | 28.6 ± 0.4 |
|  | TNF-α | 29.6 ± 0.4 | 10.8 ± 0.9 | 63.4 ± 3.3 |
